# Congenital syphilis: performance of primary care services in São Paulo, 2017

**DOI:** 10.11606/s1518-8787.2023057004965

**Published:** 2023-10-24

**Authors:** Caroline Eliane Couto, Elen Rose Lodeiro Castanheira, Patrícia Rodrigues Sanine, Carolina Siqueira Mendonça, Luceime Olívia Nunes, Thais Fernanda Tortorelli Zarili, Adriano Dias

**Affiliations:** I Universidade Estadual Paulista “Júlio de Mesquita Filho” Faculdade de Medicina de Botucatu Programa de Pós-Graduação em Saúde Coletiva Botucatu SP Brazil Universidade Estadual Paulista “Júlio de Mesquita Filho” . Faculdade de Medicina de Botucatu . Programa de Pós-Graduação em Saúde Coletiva . Botucatu , SP , Brazil; II Universidade Estadual Paulista “Júlio de Mesquita Filho” Faculdade de Medicina de Botucatu Departamento de Saúde Pública Botucatu SP Brazil Universidade Estadual Paulista “Júlio de Mesquita Filho” . Faculdade de Medicina de Botucatu . Departamento de Saúde Pública . Botucatu , SP , Brazil; III Universidade Estadual do Oeste do Paraná Centro de Ciências Biológicas e da Saúde Cascavel PR Brazil Universidade Estadual do Oeste do Paraná . Centro de Ciências Biológicas e da Saúde . Cascavel , PR , Brazil

**Keywords:** Syphilis, Congenital, Primary Health Care, Health Services Research, Disease Prevention, Surveys and Questionnaires

## Abstract

**OBJECTIVE:**

To evaluate congenital syphilis prevention actions in primary health care services in the state of São Paulo.

**METHODS:**

Cross-sectional evaluative research that used indicators extracted from the Survey of Evaluation and Monitoring of Primary Care Services ( *Avaliação e Monitoramento de Serviços da Atenção Básica* – QualiAB) in the state of São Paulo in 2017. An evaluative matrix composed of 31 indicators of prevention of congenital syphilis, categorized into four domains of analysis: diagnosis and treatment of acquired syphilis (10); basic infrastructure and resources (7); prevention of congenital syphilis during prenatal care (7); and educational actions and prevention of sexually transmitted infections (7). The frequency of services with positive responses for each indicator and the percentage of service performance were calculated based on the proportion of indicators reported per service and the overall average observed. Subsequently, services were classified into four quality groups, and associations between groups and each indicator, type of organizational arrangement and location were estimated.

**RESULTS:**

2,565 services participated, located in 503 municipalities, with an overall average performance of 74.9%. The domain “diagnosis and treatment of acquired syphilis” had the highest performance (89.8%), followed by “infrastructure and basic resources” (79.5%), “prevention of congenital syphilis in prenatal care” (73.3%) and “educational actions and prevention of sexually transmitted infections” (56.8%). There was a significant difference between quality groups and all indicators and types of organizational arrangements.

**CONCLUSIONS:**

The evaluated services have limitations in the development of actions to prevent congenital syphilis, mainly related to health education and actions included in prenatal care, such as screening and adequate treatment of pregnant women and their partners. Changes are needed in the work process, with the expansion of educational and surveillance actions, as well as the qualification of the teams to effectively comply with the protocols.

## INTRODUCTION

Despite the extensive knowledge on its prevention, the high incidence rates, complications, and deaths related to congenital syphilis maintain it as one of the main causes of child morbidity and mortality ^1^ . Worldwide data indicate that, in 2016, the occurrence of maternal syphilis caused about 355,000 adverse outcomes in pregnancies, including approximately 140,000 early fetal deaths and stillbirths, 14,000 neonatal deaths, 41,000 premature or low birth weight children and 109 thousand cases of clinical signs in newborns. In that same year, the incidence rate of congenital syphilis in the world was 4.73/1,000 live births, with 660,000 reported cases ^1^ .

Faced with the magnitude and serious repercussion of congenital syphilis on maternal and child health, the World Health Organization (WHO) established the reduction of the annual incidence rate to 0.5/1,000 live births ^2 , 3^ .

In Brazil, the goal of eliminating mother-to-child transmission of syphilis was included in the Ministry of Health’s Guide for the Certification of the Elimination of Vertical Transmission of HIV and/or Syphilis ( *Guia para Certificação da Eliminação da Transmissão Vertical de HIV e/ou Sífilis* ) in 2021 ^4^ . In 2020, 22,065 cases of congenital syphilis were reported in Brazil, corresponding to an incidence rate of 7.7/1,000 live births, with the Southeast (8.9/1,000 live births) being the region with the highest incidence rate in the country. This year, the state of São Paulo had the lowest incidence rate in the region (5.6/1,000 live births), reversing the progressive increase observed by 2018 ^5^ .

Currently, there are well-established protocols and guidelines that address the prevention of mother-to-child transmission of syphilis ^2^ . In this sense, primary health care (PHC) services are highlighted since congenital syphilis can be avoided through various actions related to prenatal care, screening, and treatment of maternal infection at this level of care ^2^ .

Considering that congenital syphilis is a preventable disease with resources available in the Brazilian Unified Health System (SUS) ^2^ , its occurrence represents a sentinel event of the quality of care provided in prenatal care ^7 , 8^ . In this sense, the high number of cases in Brazil and in the state of São Paulo ^5^ indicates the loss of the opportunity to interrupt the transmission chain in PHC, suggesting flaws in the organization of services at this level of care.

The evaluation focused on the organization of the work process ^9^ in PHC services allows identifying process variables that indicate the expected results, as proposed in the Donabedian model ^12^ . In this sense, evaluating the actions for congenital syphilis prevention allows us to understand how much the practices included in the services’ routines are close to the recommendations of national and international protocols and guidelines ^2^ .

Considering the importance of PHC in congenital syphilis prevention and the gap between accumulated knowledge and practice in services, this study aims to evaluate the organization of actions for congenital syphilis prevention in PHC services in the state of São Paulo.

## METHODS

This is an evaluative, cross-sectional study focused on the organization of actions to prevent congenital syphilis in PHC services. In it, the construction of a logical model ^13^ and an evaluation matrix defined the election of the indicators selected for the evaluation. We used secondary data from the survey carried out in the state of São Paulo, in 2017, with the Questionnaire for the Evaluation and Monitoring of Primary Care Services ( *Avaliação e Monitoramento de Servicços de Atenção Básica* – QualiAB).

The QualiAB is a validated, electronically self-applied instrument, composed of structure and process indicators of the diversified set of actions under the responsibility of PHC services ^9 , 14^ . Due to its scope, it allows the assessment of different dimensions of health care based on the performance measured in relation to the PHC guidelines in SUS ^10 , 11 , 15^ .

QualiAB was made available electronically to all São Paulo municipalities in 2017, with the support of the São Paulo State Health Department. A total of 2,739 services, located in 514 municipalities, responded the survey. Notably, the city of São Paulo did not join the survey. To ensure the representativeness of the set of services evaluated, those that reported not having prenatal care were excluded from the analysis.

For the selection of variables related to the evaluative dimension “prevention of congenital syphilis in PHC,” a matrix composed of 31 variables was developed, considered indicators of the organizational quality of services. The indicators were grouped into four domains of analysis: infrastructure and basic resources (10); educational actions and prevention of sexually transmitted infections (STIs) (7); diagnosis and treatment of acquired syphilis (7); and prevention of congenital syphilis during prenatal care (7) ( Chart ). The evaluation standards used in this study follow the recommendations of protocols and policies for the prevention and elimination of congenital syphilis ^2^ .

**Chart:** Theoretical matrix for evaluating the dimension “prevention of congenital syphilis in primary health care,” São Paulo, 2017.

Domain	Scope of actions in primary care	Indicators (n)
Infrastructure and basic resources	Guarantee of human and material resources to carry out the recommended actions, as well as the provision of basic procedures and technical training of professionals and services.	10
Educational actions and STI prevention	Health education actions aimed at encouraging autonomy in the experience of sexuality and reproduction, with individual empowerment in the participation in the health-disease process, in addition to carrying out intersectoral activities that address groups of greater vulnerability.	7
Diagnosis and treatment of acquired syphilis	Assistance for STIs, especially syphilis, with diagnosis, treatment, monitoring and follow-up of carriers, considering the importance of strategies for adherence.	7
Prenatal prevention of congenital syphilis	Prenatal care that influences the effectiveness of actions to prevent mother-to-child transmission of syphilis.	7
Total indicators of the dimension “prevention of congenital syphilis in primary health care”		31

STI: sexually transmitted infections.

For services characterization, variables related to location (rural, urban) and type of organizational arrangement (Family Health Unit – USF; Basic Health Unit – traditional UBS; UBS with Community Health Agents Program – PACS; or Family Health Strategy – ESF, and other types of arrangements) were included.

The extraction of information from the original database of the QualiAB application in 2017 allowed the construction of a database composed of variables related to the evaluative dimension “prevention of congenital syphilis in PHC.” The services’ responses to the indicators were dichotomously categorized: 1 corresponds to what the service is referred to do and 0 to what it did not refer to.

At first, the absolute and relative frequencies of services with positive responses for each of the 31 indicators were estimated, that is, the proportion of services that reported carrying out the recommended actions.

Next, the performance per service was analyzed based on the proportion of indicators achieved. The average of positive responses in each domain was initially calculated (sum of positive responses divided by the number of indicators and multiplied by 100), with the result varying, therefore, between 0% and 100% within that domain. The performance of each service in the evaluative dimension “prevention of congenital syphilis in PHC” was calculated by the average performance in the four domains (sum of the performance in the domains divided by four).

The performance percentage of the set of participating services was evaluated according to the distribution of performances per service in the evaluative dimension “prevention of congenital syphilis in PHC,” using measures of central tendency and dispersion (mean, median, and standard deviation).

In the end, the participating services were classified into quality groups (clusters), based on the distribution of performance per service in the evaluative dimension “prevention of congenital syphilis in PHC” in quartiles. Chi-square tests were used to estimate associations between each indicator and quality groups, followed by Z tests. The same strategy was used to estimate associations between quality groups and service characterization variables: type of organizational arrangement and location. Variables with p-values < 0.05 were considered statistically significant. Calculations were performed using the IBM/SPSS v. 26.0.

The study was approved by the Research Ethics Committee of the Faculdade de Medicina de Botucatu of Universidade Estadual Paulista “Júlio de Mesquita Filho” (CAAE: 83473518.1.0000.5411), under opinion no. 4,552,843, on February 23, 2021.

## RESULTS

In this study we evaluated 2,565 PHC services, located in 503 municipalities in the state of São Paulo, of which 91.5% were located in urban areas and 8.5% in rural areas. Distribution by organizational arrangement showed 47.2% of USF; 27.2% of UBS with PACS or ESF; 22.5% from traditional UBS; and 3.1% from other types of arrangements, such as UBS with emergency care or polyclinics.

Table 1 shows the frequencies of services according to each domain’s indicators of the dimension “prevention of congenital syphilis in PHC”. In the domain “infrastructure and basic resources,” 84.4% of the participating services reported offering rapid test for syphilis in the unit’s routine, but only 75.8% reported sampling for laboratory tests (blood and urine) as a routine procedure, and 73.3% reported administering benzathine penicillin. Another relevant aspect is that 65.1% of the services participated in training strategies and/or continuing education on STIs and aids in the year prior to participating in the study.

**Table 1:** Absolute and relative frequency of primary health care services in the state of São Paulo with a positive response to the indicators of the dimension “prevention of congenital syphilis in primary health care,” grouped according to domains, São Paulo, 2017 (n = 2,565).

Dimension: Prevention of congenital syphilis in primary health care – Indicators	n	%
Domain: Infrastructure and basic resources		
Availability of male condoms in the unit	2,545	99.2
Availability of a permanent nurse in the unit	2,511	97.9
Access to reference services in STI, aids and viral hepatitis	2,260	88.1
Availability of general practitioner and/or family doctor fixed in the unit	2,225	86.7
Availability of female condoms in the unit	2,222	86.6
Rapid test offer for syphilis in the unit’s routine	2,166	84.4
Sampling for laboratory tests (blood and urine) as a routine procedure in the unit	1,944	75.8
Administration of benzathine penicillin (Benzetacil) as a routine procedure in the unit	1,881	73.3
Participation in training strategies and/or continuing education on STIs and aids in the last year	1,671	65.1
Availability of the most used drugs for the treatment of STIs	969	37.8
Domain: Educational actions and STI/aids prevention		
Actions that address STIs, aids, and viral hepatitis regularly developed in adult care	2,052	80.0
STI/aids theme addressed in health education actions carried out at the unit in the last year	1,970	76.8
Guidance on sexuality and STI/aids prevention included in routine care for adolescents in the unit	1,671	65.1
Guidelines on sexuality and STI/aids prevention included in adolescent care actions developed in the community	1,515	59.1
People who abuse alcohol and other drugs as a target audience for programmatic actions to prevent STI/aids carried out by the unit	1,086	42.3
STI/aids prevention addressed in health education actions carried out in the community in the last year	1,014	39.5
More vulnerable groups (truck drivers, sex workers, among others) as a target audience for programmatic actions to prevent STI/aids carried out by the unit	886	34.5
Domain: Diagnosis and treatment of acquired syphilis		
Calling the user in case of a positive result in serology for syphilis	2,429	94.7
Guidance on care with partners in case of a positive result in serology for syphilis	2,412	94.0
Guidance on diagnosis and treatment in case of a positive result in serology for syphilis	2,404	93.7
Completion of the compulsory notification form in cases of syphilis	2,368	92.3
Performing a rapid test or serology for syphilis in a case suggestive of STI/aids	2,237	87.2
Treatment and follow-up carried out in the unit in case of a positive result in serology for syphilis	2,166	84.4
Calling users with syphilis who miss appointments	2,112	82.3
Domain: Prenatal prevention of congenital syphilis		
Six or more prenatal appointments	2,451	95.6
Calling pregnant women who miss prenatal appointments	2,370	92.4
Offer of syphilis treatment for pregnant women and partner in the unit	1,994	77.7
Mean proportion of pregnant women with prenatal care initiated in the first trimester greater than 80%	1,986	77.4
Request for rapid test or serology for syphilis in the first and third trimesters of pregnancy	1,791	69.8
Men’s prenatal care	1,294	50.4
Absence of cases of congenital syphilis in the unit in the last three years	1,277	49.8

STI: sexually transmitted infections.

As for “educational actions and STI prevention,” a predominance of actions that address STIs, aids, and viral hepatitis developed regularly in adult care in 80% of the services, and the STI/aids theme in education actions performed at the unit in 76.8%, were highlights. STI/aids prevention addressed in community health education actions was little explored, being reported by 39.5%. People who abuse alcohol and other drugs, as well as vulnerable groups, were not a frequent target audience for STI/aids prevention actions carried out by the units, only in 42.3% and 34.5%, respectively ( Table 1 ).

Regarding the domain “diagnosis and treatment of acquired syphilis,” 94% of the services reported providing guidance on care for sexual partners and 93.7% guidance on diagnosis and treatment. However, only 84.4% reported treating and following up these cases in the unit itself ( Table 1 ).

In the domain “prevention of congenital syphilis in prenatal care,” recommended strategies, such as carrying out six or more prenatal care visits and calling pregnant women who miss appointments, were followed by most services, with a frequency of 95.6% and 92.4%, respectively. The offer of syphilis treatment for pregnant women and their partners in the unit was mentioned by 77.7% of the services, and the request for rapid test or serology for syphilis in the first and third trimester of pregnancy was mentioned by 69.8%. The absence of cases of congenital syphilis in the area covered by the unit in the three years prior to the assessment was reported by 49.8% ( Table 1 ).

Table 2 shows that in the dimension “prevention of congenital syphilis in PHC,” the services presented a mean performance of 74.9% against the expected standards. Regarding the mean performance per domain, “diagnosis and treatment of acquired syphilis” obtained the highest percentage, with 89.8%, followed by “infrastructure and basic resources” and “prevention of congenital syphilis in prenatal care,” with 79.5% and 73.3%, respectively. The domain “educational actions and STI prevention” had a significantly lower performance than the others, with 56.8%.

**Table 2:** Percentage of performance of primary health care services in the dimension “prevention of congenital syphilis in primary health care” and subsequent domains, São Paulo, 2017 (n = 2,565).

Indicator groups	Median	Mean	SD	95%CI (Mean)
Dimension “prevention of congenital syphilis in primary health care”	76.8	74.9	14.3	74.3–75.4
Domains				
Infrastructure and basic resources	80.0	79.5	15.2	78.9–80.1
Educational actions and STI prevention	57.1	56.8	29.4	55.6–57.9
Diagnosis and treatment of acquired syphilis	100	89.8	17.7	89.1–90.4
Prenatal prevention of congenital syphilis	71.4	73.3	17.5	72.6–73.9

STI: sexually transmitted infections; SD: standard deviation; 95%CI: 95% confidence interval.

Based on the distribution of the performance of the set of services in the dimension “prevention of congenital syphilis in PHC,” quality groups were constructed to analyze the variation in performance across services ( Table 3 ). G4 gathered those services that had the highest performance (≥ 86.4%), followed by G3 (77.1–85.7%) and G2 (67.1–76.8%). The services with the lowest performance were gathered in G1 (≤ 66.4%).

**Table 3:** Quality groups (clusters) in the dimension “prevention of congenital syphilis in primary health care” by frequency and percentage of services, performance percentage range, and measures of central tendency and dispersion (n = 2,565).

Groups*	n	%	Minimum performance	Maximum performance	Mean	Median	SD
G1	675	26.3	8.6	66.4	55.9	58.9	10.0
G2	609	23.7	67.1	76.8	72.5	73.2	2.8
G3	683	26.6	77.1	85.7	81.3	80.7	2.6
G4	598	23.3	86.4	100	91.4	90.4	3.7
Total	2,565	100	-	-	-	-	-

SD: standard deviation.

*Quartile.; SD: standard deviation.

Considering the quality groups, the four groups for each indicator used in the evaluation matrix were compared ( Table 4 ). According to the chi-square test, there was a significant difference between the quality groups for all indicators. In order to identify which groups differed from each other, the Z test was applied and it showed that the proportion of almost all indicators differs across the four groups, with some exceptions: in indicators with higher frequencies in the set of services, the proportion per group tends to have similarities in G2, G3, and G4—with variations between them—however, the group with the lowest quality (G1) always differs from the others. In just two indicators (absence of cases of congenital syphilis and availability of female condoms), G1 is similar to G2.

**Table 4:** Comparison of the frequency of each indicator by the four quality groups, according to the chi-square test and Z tests (n = 2,565).

Indicators	Quality groups (clusters)								p ^**^

	G1		G2		G3		G4		
			
	n ^*^	%	n ^*^	%	n ^*^	%	n ^*^	%	
Domain: Infrastructure and basic resources									
Availability of male condoms in the unit	661 ^a^	97.9	606 ^b^	99.5	681 ^b^	99.7	597 ^b^	99.8	< 0.001
Availability of a permanent nurse in the unit	641 ^a^	95.0	603 ^b^	99.0	673 ^b^	98.5	594 ^b^	99.3	< 0.001
Access to reference services in STI, aids and viral hepatitis	526 ^a^	77.9	533 ^b^	87.5	622 ^c^	91.1	579 ^d^	96.8	< 0.001
Availability of general practitioner and/or family doctor fixed in the unit	521 ^a^	77.2	517 ^b^	84.9	618 ^c^	90.5	569 ^d^	95.2	< 0.001
Availability of female condoms in the unit	544 ^a^	80.6	515 ^a.b^	84.6	600 ^b^	87.8	563 ^c^	94.1	< 0.001
Rapid test offer for syphilis in the unit’s routine	439 ^a^	65.0	521 ^b^	85.6	622 ^c^	91.1	584 ^d^	97.7	< 0.001
Sampling for laboratory tests (blood and urine) as a routine procedure in the unit	423 ^a^	62.7	461 ^b^	75.7	541 ^b^	79.2	519 ^c^	86.8	< 0.001
Administration of benzathine penicillin (Benzetacil) as a routine procedure in the unit	345 ^a^	51.1	437 ^b^	71.8	551 ^c^	80.7	548 ^d^	91.6	< 0.001
Participation in training strategies and/or continuing education on STIs and aids in the last year	246 ^a^	36.4	374 ^b^	61.4	506 ^c^	74.1	545 ^d^	91.1	< 0.001
Availability of the most used drugs for STIs treatment	126 ^a^	18.7	198 ^b^	32.5	302 ^c^	44.2	343 ^d^	57.4	< 0.001
Educational actions and STI/aids prevention									
STI/aids theme addressed in health education actions carried out at the unit in the last year	319 ^a^	47.3	458 ^b^	75.2	605 ^c^	88.6	588 ^d^	98.3	< 0.001
Guidelines on sexuality and STI/aids prevention included in adolescent care actions developed in the community	177 ^a^	26.2	288 ^b^	47.3	487 ^c^	71.3	563 ^d^	94.1	< 0.001
Actions that address STIs, aids and viral hepatitis regularly developed in adult care	334 ^a^	49.5	487 ^b^	80.0	643 ^c^	94.1	588 ^d^	98.3	< 0.001
Guidance on sexuality and STI/aids prevention included in routine care for adolescents in the unit	198 ^a^	29.3	334 ^b^	54.8	559 ^c^	81.8	580 ^d^	97.0	< 0.001
People who abuse alcohol and other drugs as a target audience for programmatic actions to prevent STI/aids carried out by the unit	52 ^a^	7.7	149 ^b^	24.5	359 ^c^	52.6	526 ^d^	88.0	< 0.001
STI/aids prevention addressed in health education actions carried out in the community in the last year	118 ^a^	17.5	162 ^b^	26.6	305 ^c^	44.7	429 ^d^	71.7	< 0.001
More vulnerable groups (truck drivers, sex workers, among others) as a target audience for programmatic actions to prevent STI/aids carried out by the unit	34 ^a^	5.0	117 ^b^	19.2	274 ^c^	40.1	461 ^d^	77.1	< 0.001
Diagnosis and treatment of acquired syphilis									
Calling the user in case of a positive result in serology for syphilis	576 ^a^	85.3	589 ^b^	96.7	670 ^b.c^	98.1	594 ^c^	99.3	< 0.001
Guidance on diagnosis and treatment in case of a positive result in serology for syphilis	546 ^a^	80.9	587 ^b^	96.4	673 ^c^	98.5	598 ^d^	100	< 0.001
Completion of the compulsory notification form in cases of syphilis	538 ^a^	79.7	585 ^b^	96.1	658 ^b^	96.3	587 ^c^	98.2	< 0.001
Performing a rapid test or serology for syphilis in a case suggestive of STI/aids	431 ^a^	63.9	558 ^b^	91.6	656 ^c^	96.0	592 ^d^	99.0	< 0.001
Calling users with syphilis who miss appointments	364 ^a^	53.9	528 ^b^	86.7	630 ^c^	92.2	590 ^d^	98.7	< 0.001
Guidance on care with partners in case of a positive result in serology for syphilis	557 ^a^	82.5	588 ^b^	96.6	672 ^c^	98.4	595 ^c^	99.5	< 0.001
Treatment and follow-up carried out in the unit itself in case of a positive result in serology for syphilis	416 ^a^	61.6	520 ^b^	85.4	643 ^c^	94.1	587 ^d^	98.2	< 0.001
Prenatal prevention of congenital syphilis									
Six or more prenatal appointments	611 ^a^	90.5	582 ^b^	95.6	667 ^c^	97.7	591 ^c^	98.8	< 0.001
Calling pregnant women who miss prenatal appointments	531 ^a^	78.7	581 ^b^	95.4	665 ^b^	97.4	593 ^c^	99.2	< 0.001
Average proportion of pregnant women with prenatal care initiated in the first trimester greater than 80%	446 ^a^	66.1	462 ^b^	75.9	544 ^b^	79.6	534 ^c^	89.3	< 0.001
Request for rapid test or serology for syphilis in the first and third trimesters of pregnancy	339 ^a^	50.2	444 ^b^	72.9	503 ^b^	73.6	505 ^c^	84.4	< 0.001
Offer of syphilis treatment for pregnant women and partner in the unit	363 ^a^	53.8	464 ^b^	76.2	588 ^c^	86.1	579 ^d^	96.8	< 0.001
Men’s prenatal care	193 ^a^	28.6	265 ^b^	43.5	386 ^c^	56.5	450 ^d^	75.3	< 0.001
Absence of cases of congenital syphilis in the last three years	285 ^a^	42.2	279 ^a^	45.8	352 ^b^	51.5	361 ^c^	60.4	< 0.001

STI: sexually transmitted infections.

* Z tests.

** Chi-square test.

Different subscripts indicate statistically different proportions.

The crossing between the four quality groups and the types of organizational arrangements (USF, traditional UBS, UBS with PACS/ESF and others) showed a significant difference in the chi-square test (p < 0.001). Regarding the USF, the best quality group (G4) was the one with the highest proportion of these services in its composition (G4 = 56.7%; G3 = 49.6%; G2 = 45.6%; G1 = 37, 6%), and almost all groups differed from each other, except for G2 and G3, which were similar, according to the Z test.

In the case of traditional UBS, the group with the lowest quality (G1) was the one with the highest proportion (G1 = 35%; G2 = 25.6%; G3 = 18.7%; G4 = 9.7%), with all groups differing from each other. Mixed units, although distributed heterogeneously across the four groups, also have a higher proportion in the higher quality group (G4 = 31.4%; G3 = 28.4%; G2 = 25.6%; G1 = 23.6%). It is worth mentioning that, when comparing the quality groups and the service location variables, the chi-square test did not show a significant association.

## DISCUSSION

The evaluation carried out, although based on descriptive data, allowed identifying some of the main points of the work process that require qualification to strengthen the prevention of congenital syphilis in the PHC of the state of São Paulo, such as screening for syphilis in prenatal care, adequate treatment of gestational and acquired syphilis with the use of benzathine penicillin, and the incorporation of health education actions in the unit and in the territory.

The average performance of the evaluated services reveals that, although the prevention of congenital syphilis relies on actions that belong to the PHC work routine, there are still many services that fail to adequately comply with the recommendations established by protocols and guidelines, as pointed out by other studies ^15 , 16^ . Although the data refer to a survey carried out in 2017, the incidence rates of congenital syphilis in the state of São Paulo ^5^ indicate the likely persistence of the identified issues. The changes that were introduced after the covid-19 pandemic altered the routine of PHC services, compromising programmatic actions, such as prenatal care and health surveillance, which may have contributed to the maintenance of the points identified in 2017 ^17 , 18^ .

The Brazilian protocols that address the prevention of congenital syphilis emphasize the importance of prenatal care since it is during the follow-up of the pregnant woman in PHC that actions are developed toward the early diagnosis of maternal infection and its timely treatment ^4 , 6^ . However, the literature shows that, among the notified cases of congenital syphilis, a large number of mothers did receive prenatal care ^16 , 19 , 20^ , indicating that simple access to consultations has not guaranteed a reduction in the incidence rate ^21^ .

The challenge of preventing mother-to-child transmission of syphilis in Brazil is mainly centered on the compromising of the quality of care offered. The results presented here bring data that corroborate this hypothesis, since the domain “prevention of congenital syphilis in prenatal care” had the second lowest performance, that is, even in actions prioritized exhaustively by the protocols, such as screening and early treatment of gestational syphilis, there are important gaps both in relation to the offer and in the availability of inputs for its implementation.

Screening for syphilis is recommended at two moments during prenatal care in PHC: in the first and third trimesters of pregnancy ^2^ . Among the services analyzed, few followed this recommendation (69.8%), limiting themselves to requesting only one test for syphilis during the first trimester (95.3%). This highlights the difficulty of services in adapting their routine to the recommendations and even the lack of knowledge of professionals and managers regarding protocols, impact, and cost-effectiveness of adequate screening ^22^ .

Clinical protocols and guidelines act as essential tools to guide and support the practices carried out by health services, but their existence alone does not guarantee the quality of care. Factors involving the units’ organization, such as the management model and, mainly, the operational work process, can bring technical-operational limitations, impairing the quality and effectiveness of health care ^23^ .

The rapid test for syphilis is one of the screening strategies highlighted as an easy-to-perform and highly cost-effective resource. When carried out in the first appointment with the pregnant woman, it provides early diagnosis and timely treatment of maternal infection ^2^ . This has been a tool used by most of the services studied and needs continuous strengthening and encouragement for its implementation.

Difficulties in the prevention of congenital syphilis in PHC extend to the problem of using benzathine penicillin as the first-choice drug in the treatment of gestational syphilis, often sustained by the professionals’ lack of knowledge about the most appropriate therapeutic schemes ^22^ and fear of adverse reactions from its administration ^24^ .

It is the only drug capable of crossing the placental barrier and reaching the fetus, therefore, the existing weaknesses in its offer as a routine procedure for the treatment of pregnant women and partners, put access to safe, effective, and timely treatment of syphilis during pregnancy at risk ^3^ . This difficulty is sustained in this study, with indicators showing that a significant portion of the units do not offer treatment to pregnant women and partners and do not administer benzathine penicillin as a routine procedure.

Despite the insufficient performance expressed by treatment indicators, it is important to emphasize that there have been advances. When comparing results of this study with that conducted by Sanine et al. ^15^ , a better performance is observed in the indicators related to administration of benzathine penicillin in the unit and in the provision of treatment for pregnant women and partners, in 2017 compared to 2010. This improvement possibly reflects the training efforts and technical measures undertaken by both the Ministry of Health ^6 , 25 , 26^ and by the São Paulo State Health Department ^27^ .

In addition to prenatal care, the evaluation model for the prevention of congenital syphilis presented considers the importance of practices that favor the interruption of the syphilis transmission chain in other life stages through prevention actions, early diagnosis, and management of syphilis in women of childbearing age and their sexual partners.

The evaluated services have a good performance related to STI care, and the domain “diagnosis and treatment of acquired syphilis” was the one that obtained the highest mean among the services, and none of its indicators had a frequency lower than 80%. This result may be a reflection of the tradition of assistance to STIs in PHC in São Paulo, since the state was a pioneer in organizing the network’s responses to the aids epidemic in the country and, since then, has assumed a strong role in the line of prevention and assistance to STIs/aids, undertaking initiatives that aim to strengthen the integration of these actions ^27 , 28^ .

In the search for the elimination of congenital syphilis, it is important that educational actions are carried out with the community, pregnant women and families, with the aim of increasing these actors’ knowledge about maternal and child health and guaranteeing greater autonomy in care ^4^ . However, the domain “educational actions and STI prevention” was the one with the lowest performance, with low frequencies in all indicators, especially in those that address health education practices developed in the community and that involve vulnerable groups. These results corroborate the literature, which indicates that STI/aids prevention practices and health education occupy a secondary place in the health work process ^10 , 29^ .

The high and similar means and medians between the quality groups indicate the occurrence of a small percentage interval between them, with the exception of G1, indicating that about 70% of the services had an average performance above 70%. However, for a disease that is preventable through different practices developed in PHC services at different times, this performance can be considered insufficient.

All the indicators included in the evaluation matrix were capable of differentiating the groups from each other, a fact that can be statistically justified by the large number of services in the sample (2,565), but, on the other hand, reflects the differentiation between the services and the capacity of the matrix proposal to identify distinct attributes that qualify the prevention of congenital syphilis.

The better performance of units organized according to family health guidelines is well demonstrated in the literature ^30^ , and the evaluation carried out reaffirms this tendency. On the other hand, the USF and the UBS with integrated ESF or PACS also require a greater investment in order to provide comprehensive care since many of these units also showed low performance.

One of the limits of this work is the generalizability of the results since they refer only to the participating services. Another limit concerns the use of secondary data from a survey that evaluated the set of PHC actions, with the prevention of congenital syphilis being a part of this set. In addition, descriptive cross-sectional studies have limits given by the time frame they make of reality. However, the evaluation of service performance points out issues that require procedural changes and planning and management measures, contributing to quality improvement.

The weaknesses identified in the organization of services have great local governance and point to priorities that need to be incorporated and that represent the consolidation of policies and guidelines already available. The need for investments in the technical capacity of the services and in professional qualification for the effective execution of the protocols is highlighted.

The results confirm the importance of qualifying prenatal care, already pointed out in other studies, and highlight the value of carrying out assessments that induce reflections in the teams on the organization of work processes and, thus, contribute to the establishment of change processes that have an effect on the prevention of congenital syphilis.
