# Supplementary material for: Congenital syphilis: performance of primary care services in São Paulo, 2017
Source: Rev Saude Publica. 2023 Oct 24;57:78. doi: 10.11606/s1518-8787.2023057004965 (PMC10609639; doi:10.11606/s1518-8787.2023057004965)
Supplement: Translation

# Translation: Sífilis congênita: desempenho de serviços da atenção primária paulista, 2017

**Authors:** Caroline Eliane Couto, Elen Rose Lodeiro Castanheira, Patrícia Rodrigues Sanine, Carolina Siqueira Mendonça, Luceime Olívia Nunes, Thais Fernanda Tortorelli Zarili, Adriano Dias

**Affiliations:** I Brasil Universidade Estadual Paulista “Júlio de Mesquita Filho”. Faculdade de Medicina de Botucatu. Programa de Pós-Graduação em Saúde Coletiva. Botucatu, SP, Brasil; II Brasil Universidade Estadual Paulista “Júlio de Mesquita Filho”. Faculdade de Medicina de Botucatu. Departamento de Saúde Pública. Botucatu, SP, Brasil; III Brasil Universidade Estadual do Oeste do Paraná. Centro de Ciências Biológicas e da Saúde. Cascavel, PR, Brasil

**Keywords:** Sífilis Congênita, Atenção Primária à Saúde, Pesquisa sobre Serviços de Saúde, Prevenção de Doenças, Inquéritos e Questionários

## Abstract

**OBJETIVO:**

Avaliar as ações de prevenção da sífilis congênita em serviços de atenção primária à saúde no estado de São Paulo.

**MÉTODOS:**

Pesquisa avaliativa transversal que utilizou indicadores extraídos da aplicação do inquérito de Avaliação e Monitoramento de Serviços de Atenção Básica (QualiAB) no estado de São Paulo em 2017. Foi construída uma matriz avaliativa composta de 31 indicadores de prevenção da sífilis congênita, categorizados em quatro domínios de análise: diagnóstico e tratamento da sífilis adquirida (10); infraestrutura e recursos básicos (7); prevenção da sífilis congênita no pré-natal (7); e ações educativas e prevenção de infecções sexualmente transmissíveis (7). Calculou-se a frequência dos serviços com respostas positivas para cada indicador e o percentual de desempenho dos serviços a partir da proporção de indicadores referida por serviço e da média geral observada. Posteriormente, os serviços foram classificados em quatro grupos de qualidade e foram estimadas associações entre os grupos e cada indicador, tipo de arranjo organizacional e localização.

**RESULTADOS:**

Participaram 2.565 serviços, localizados em 503 municípios, com média geral de desempenho de 74,9%. O domínio “diagnóstico e tratamento da sífilis adquirida” obteve maior desempenho (89,8%), seguido de “infraestrutura e recursos básicos” (79,5%), “prevenção da sífilis congênita no pré-natal” (73,3%) e “ações educativas e prevenção de infecções sexualmente transmissíveis” (56,8%). Observou-se uma diferença significativa entre os grupos de qualidade e todos os indicadores e tipos de arranjos organizacionais.

**CONCLUSÕES:**

Os serviços avaliados possuem limitações no desenvolvimento das ações de prevenção da sífilis congênita, principalmente relacionadas à educação em saúde e às ações inseridas no acompanhamento pré-natal, como rastreio e tratamento adequado da gestante e sua parceria. São necessárias mudanças no processo de trabalho, com a ampliação de ações educativas e de vigilância, assim como a qualificação das equipes para o cumprimento dos protocolos de maneira efetiva.

## INTRODUÇÃO

Apesar do extenso conhecimento sobre a prevenção da sífilis congênita, as altas taxas de incidência, as complicações e os óbitos relacionados a esse agravo a mantêm como uma das principais causas de morbimortalidade infantil^1^. Dados mundiais apontam que, em 2016, a ocorrência de sífilis materna causou cerca de 355 mil resultados adversos em gestações, entre os quais estão aproximadamente 140 mil óbitos fetais precoces e natimortos, 14 mil óbitos neonatais, 41 mil crianças prematuras ou com baixo peso e 109 mil casos de sinais clínicos em recém-nascidos. Nesse mesmo ano, a taxa de incidência de sífilis congênita no mundo foi de 4,73/1.000 nascidos vivos, com 660 mil casos notificados^1^.

Diante da magnitude e grave repercussão da sífilis congênita na saúde materno-infantil, a Organização Mundial da Saúde (OMS) estabeleceu como meta para a eliminação da sífilis congênita como um problema de saúde pública a redução da taxa de incidência anual para 0,5/1.000 nascidos vivos^2 , 3^.

No Brasil, a meta de eliminação da transmissão vertical da sífilis foi incluída no Guia para Certificação da Eliminação da Transmissão Vertical de HIV e/ou Sífilis, do Ministério da Saúde, em 2021^4^. Em 2020, foram notificados 22.065 casos de sífilis congênita no Brasil, que correspondem a uma taxa de incidência de 7,7/1.000 nascidos vivos, sendo o Sudeste (8,9/1.000 nascidos vivos) a região com a maior taxa de incidência no país. Neste ano, o estado de São Paulo apresentou a menor taxa de incidência da região (5,6/1.000 nascidos vivos), invertendo a tendência de aumento progressivo verificada até 2018^5^.

Atualmente há protocolos e diretrizes bem estabelecidos que abordam a prevenção da transmissão vertical da sífilis^2^. Nesse sentido, os serviços de atenção primária à saúde (APS) recebem destaque, pois a sífilis congênita pode ser evitada por meio de diversas ações relacionadas à atenção pré-natal, ao rastreamento e ao tratamento da infecção materna neste nível de atenção^2^.

Considerando que a sífilis congênita é uma doença evitável com recursos disponíveis no Sistema Único de Saúde (SUS)^2^, sua ocorrência representa um evento sentinela da qualidade da assistência prestada no pré-natal^7 , 8^. Nesse sentido, o elevado número de casos no Brasil e no estado de São Paulo^5^indica a perda da oportunidade de interromper a cadeia de transmissão na APS, sugerindo falhas na organização dos serviços desse nível de atenção.

A avaliação com foco na organização do processo de trabalho^9^em serviços de APS permite identificar variáveis de processo que indicam os resultados esperados, como proposto no modelo donabediano^12^. Nesse sentido, avaliar as ações de prevenção da sífilis congênita permite compreender o quanto as práticas inseridas nas rotinas dos serviços se aproximam das recomendações de protocolos e diretrizes nacionais e internacionais^2^.

Considerando a importância da APS na prevenção da sífilis congênita e a lacuna entre o conhecimento acumulado e a prática nos serviços, este estudo teve como objetivo avaliar a organização de ações de prevenção da sífilis congênita em serviços de APS do estado de São Paulo.

## MÉTODOS

Trata-se de uma pesquisa avaliativa, de corte transversal e com foco na organização das ações de prevenção à sífilis congênita em serviços de APS. Nela, a construção de um modelo lógico^13^e uma matriz avaliativa definiram a eleição dos indicadores selecionados para a avaliação. Foram utilizados dados secundários do inquérito realizado no estado de São Paulo, em 2017, com o Questionário de Avaliação e Monitoramento de Serviços de Atenção Básica (QualiAB).

O QualiAB é um instrumento validado, autoaplicado eletronicamente, composto de indicadores de estrutura e processo do conjunto diversificado de ações sob responsabilidade dos serviços de APS^9 , 14^. Por sua abrangência, permite a avaliação de diferentes dimensões da atenção à saúde a partir do desempenho mensurado em relação às diretrizes da APS no SUS^10 , 11 , 15^.

O QualiAB foi disponibilizado eletronicamente para todos os municípios paulistas, em 2017, com o apoio da Secretaria de Saúde do Estado de São Paulo. Responderam ao inquérito 2.739 serviços, localizados em 514 municípios. Cabe aqui destacar que não houve adesão do município de São Paulo ao inquérito. Para garantir a representatividade do conjunto de serviços avaliados, foram excluídos da análise aqueles que informaram não realizar acompanhamento pré-natal.

Para a seleção das variáveis relacionadas à dimensão avaliativa “prevenção da sífilis congênita na APS”, foi desenvolvida uma matriz composta de 31 variáveis, consideradas indicadores da qualidade organizacional dos serviços. Os indicadores foram reunidos em quatro domínios de análise: infraestrutura e recursos básicos (10); ações educativas e prevenção de infecções sexualmente transmissíveis (IST) (7); diagnóstico e tratamento da sífilis adquirida (7); e prevenção da sífilis congênita no pré-natal (7) ( Quadro ). Os padrões avaliativos utilizados neste estudo seguem as recomendações de protocolos e políticas de prevenção e eliminação da sífilis congênita^2^.

**Quadro:** Matriz teórica para avaliação da dimensão “prevenção da sífilis congênita na atenção primária à saúde”, São Paulo, 2017.

Domínio	Escopo das ações na atenção primária	Indicadores (n)
Infraestrutura e recursos básicos	Garantia de recursos humanos e materiais para a realização das ações preconizadas, assim como a oferta de procedimentos básicos e a capacitação técnica de profissionais e serviços.	10
Ações educativas e prevenção de IST	Ações de educação em saúde voltadas ao incentivo à autonomia na vivência da sexualidade e da reprodução, com empoderamento individual na participação do processo de saúde-doença, além de realização de atividades intersetoriais que abordam grupos de maior vulnerabilidade.	7
Diagnóstico e tratamento da sífilis adquirida	Assistência às IST, em especial à sífilis, com realização de diagnóstico, tratamento, monitoramento e acompanhamento de indivíduos portadores, considerando a importância de estratégias para a adesão.	7
Prevenção da sífilis congênita no pré-natal	Cuidados pré-natais que influenciam a efetividade das ações de prevenção da transmissão vertical da sífilis.	7
Total de indicadores da dimensão “prevenção da sífilis congênita na atenção primária à saúde”		31

IST: infecções sexualmente transmissíveis.

Para a caracterização dos serviços, foram incluídas variáveis referentes à localização (rural, urbana) e ao tipo de arranjo organizacional (Unidade de Saúde da Família – USF, Unidade Básica de Saúde – UBS tradicional, UBS com Programa de Agentes Comunitários de Saúde – PACS ou Estratégia Saúde da Família – ESF, e outros tipos de arranjos).

A extração de informações do banco original da aplicação do QualiAB em 2017 permitiu a construção de uma base composta por variáveis relativas à dimensão avaliativa “prevenção da sífilis congênita na APS”. As respostas dos serviços aos indicadores foram dicotomicamente categorizadas: 1 corresponde ao que o serviço referia fazer e 0 ao que não referia.

Em um primeiro momento, foram estimadas as frequências absolutas e relativas de serviços com respostas positivas para cada um dos 31 indicadores, ou seja, a proporção de serviços que referem realizar as ações preconizadas.

A seguir, foi analisado o desempenho por serviço a partir da proporção de indicadores alcançados. Calculou-se inicialmente a média das respostas positivas em cada domínio (soma das respostas positivas dividida pelo número de indicadores e multiplicada por 100), com o resultado variando, portanto, entre 0% e 100% dentro daquele domínio. O desempenho de cada serviço na dimensão avaliativa “prevenção da sífilis congênita na APS” foi calculado pela média do desempenho nos quatro domínios (soma do desempenho nos domínios dividida por quatro).

O percentual de desempenho do conjunto de serviços participantes foi avaliado segundo a distribuição dos desempenhos por serviço na dimensão avaliativa “prevenção da sífilis congênita na APS”, por meio de medidas de tendência central e dispersão (média, mediana e desvio padrão).

Ao final, os serviços participantes foram classificados em grupos de qualidade ( *clusters* ), com base na distribuição do desempenho por serviço na dimensão avaliativa “prevenção da sífilis congênita na APS” em quartis. Foram utilizados os testes qui-quadrado para estimar associações entre cada indicador e os grupos de qualidade, seguidos por testes Z. A mesma estratégia foi utilizada para estimar associações entre os grupos de qualidade e as variáveis de caracterização dos serviços: tipo de arranjo organizacional e localização. Foram consideradas estatisticamente significativas as variáveis com valores-p < 0,05. Os cálculos foram realizados utilizando o pacote IBM/SPSS v. 26.0.

O estudo foi aprovado pelo Comitê de Ética em Pesquisa da Faculdade de Medicina de Botucatu da Universidade Estadual Paulista “Júlio de Mesquita Filho” (CAAE: 83473518.1.0000.5411), sob o parecer nº 4.552.843, em 23 de fevereiro de 2021.

## RESULTADOS

Foram avaliados neste estudo 2.565 serviços de APS, localizados em 503 municípios paulistas, dos quais 91,5% estavam localizados em zona urbana e 8,5% em zona rural. A distribuição por arranjo organizacional mostrou 47,2% de USF; 27,2% de UBS com PACS ou ESF; 22,5% de UBS tradicionais; e 3,1% de outros tipos de arranjos, como UBS com pronto atendimento ou policlínicas.

As frequências dos serviços segundo os indicadores de cada domínio da dimensão “prevenção da sífilis congênita na APS” são apresentadas na Tabela 1 . No domínio “infraestrutura e recursos básicos”, observa-se que 84,4% dos serviços participantes relataram ofertar teste rápido para sífilis na rotina da unidade, porém, apenas 75,8% citaram coletar material para exames laboratoriais (sangue e urina) como procedimento de rotina e 73,3% informaram aplicar penicilina benzatina. Outro aspecto relevante é que 65,1% dos serviços participaram de estratégias de formação e/ou educação permanente sobre IST e aids no ano anterior à participação no estudo.

**Tabela 1:** Frequência absoluta e relativa de serviços de atenção primária à saúde do estado de São Paulo com resposta positiva aos indicadores da dimensão “prevenção da sífilis congênita na atenção primária à saúde”, agrupados segundo domínios, São Paulo, 2017 (n = 2.565).

Dimensão: Prevenção da sífilis congênita na atenção primária à saúde – Indicadores	n	%
Domínio: Infraestrutura e recursos básicos		
Disponibilidade de preservativo masculino na unidade	2.545	99,2
Disponibilidade de enfermeiro fixo na unidade	2.511	97,9
Acesso a serviços de referência em IST, aids e hepatites virais	2.260	88,1
Disponibilidade de clínico geral e/ou médico de família fixo na unidade	2.225	86,7
Disponibilidade de preservativo feminino na unidade	2.222	86,6
Oferta de teste rápido para sífilis na rotina da unidade	2.166	84,4
Coleta de material para exames laboratoriais (sangue e urina) como procedimento de rotina na unidade	1.944	75,8
Aplicação de penicilina benzatina (Benzetacil) como procedimento de rotina na unidade	1.881	73,3
Participação em estratégias de formação e/ou educação permanente sobre IST e aids no último ano	1.671	65,1
Disponibilidade dos medicamentos para o tratamento de IST mais utilizados	969	37,8
Domínio: Ações educativas e prevenção de IST/aids		
Ações que abordam IST, aids e hepatites virais desenvolvidas com regularidade na atenção ao adulto	2.052	80,0
Tema IST/aids abordado em ações de educação em saúde realizadas na unidade no último ano	1.970	76,8
Orientações sobre sexualidade e prevenção de IST/aids incluídas na rotina da atenção ao adolescente na unidade	1.671	65,1
Orientações sobre sexualidade e prevenção de IST/aids incluídas em ações para atenção ao adolescente desenvolvidas na comunidade	1.515	59,1
Pessoas que fazem uso abusivo de álcool e outras drogas como público-alvo de ações programáticas de prevenção de IST/aids realizadas pela unidade	1.086	42,3
Prevenção de IST/aids abordada em ações de educação em saúde realizadas na comunidade no último ano	1.014	39,5
Grupos mais vulneráveis (caminhoneiros, profissionais do sexo, entre outros) como público-alvo das ações programáticas de prevenção de IST/aids realizadas pela unidade	886	34,5
Domínio: Diagnóstico e tratamento da sífilis adquirida		
Convocação de usuário em caso de resultado positivo em sorologia para sífilis	2.429	94,7
Orientação de cuidado com as parcerias em caso de resultado positivo em sorologia para sífilis	2.412	94,0
Orientação sobre diagnóstico e tratamento em caso de resultado positivo em sorologia para sífilis	2.404	93,7
Preenchimento da ficha de notificação compulsória em casos de sífilis	2.368	92,3
Realização de teste rápido ou sorologia para sífilis em caso sugestivo de IST/aids	2.237	87,2
Tratamento e seguimento realizado na própria unidade em caso de resultado positivo em sorologia para sífilis	2.166	84,4
Convocação de usuários faltosos com sífilis	2.112	82,3
Domínio: Prevenção da sífilis congênita no pré-natal		
Realização de seis ou mais consultas de pré-natal	2.451	95,6
Convocação de gestantes faltosas	2.370	92,4
Oferta de tratamento da sífilis para gestante e parceria na unidade	1.994	77,7
Proporção média de gestantes com pré-natal iniciado no primeiro trimestre superior a 80%	1.986	77,4
Solicitação de teste rápido ou sorologia para sífilis no primeiro e terceiro trimestre gestacional	1.791	69,8
Realização do pré-natal do homem	1.294	50,4
Ausência de casos de sífilis congênita na unidade nos últimos três anos	1.277	49,8

IST: infecções sexualmente transmissíveis.

Quanto às “ações educativas e prevenção de IST”, destacou-se o predomínio de ações que abordam IST, aids e hepatites virais desenvolvidas com regularidade na atenção ao adulto em 80% dos serviços, e do tema IST/aids em ações de educação em saúde realizadas na unidade em 76,8%. A prevenção de IST/aids abordada em ações de educação em saúde realizadas na comunidade foi pouco explorada, sendo relatada por 39,5%. Pessoas que fazem uso abusivo de álcool e outras drogas, assim como grupos em situação de vulnerabilidade, não foram público-alvo frequente das ações de prevenção de IST/aids realizadas pelas unidades, apenas de 42,3% e 34,5%, respectivamente ( Tabela 1 ).

No que diz respeito ao domínio “diagnóstico e tratamento da sífilis adquirida”, 94% dos serviços relataram realizar orientações sobre cuidados com as parcerias sexuais e 93,7% orientações sobre diagnóstico e tratamento. Entretanto, somente 84,4% afirmaram fazer o tratamento e seguimento desses casos na própria unidade ( Tabela 1 ).

No domínio “prevenção da sífilis congênita no pré-natal”, estratégias preconizadas, como a realização de seis ou mais consultas durante o pré-natal e a convocação de gestantes faltosas, foram seguidas pela maioria dos serviços, com frequência de 95,6% e 92,4%, respectivamente. A oferta do tratamento da sífilis para gestante e parceria na unidade foi citada por 77,7% dos serviços, e a solicitação de teste rápido ou sorologia para sífilis no primeiro e terceiro trimestre gestacional foi referida por 69,8%. A ausência de casos de sífilis congênita na área de abrangência da unidade nos três anos anteriores à avaliação foi relata por 49,8% ( Tabela 1 ).

Na Tabela 2 é possível observar que na dimensão “prevenção da sífilis congênita na APS” os serviços apresentaram um desempenho médio de 74,9% em relação aos padrões esperados. Em relação ao desempenho médio por domínio, o “diagnóstico e tratamento da sífilis adquirida” foi o que obteve maior percentual, com 89,8%, seguido de “infraestrutura e recursos básicos” e “prevenção da sífilis congênita no pré-natal”, com 79,5% e 73,3%, respectivamente. Destaca-se que o domínio “ações educativas e prevenção de IST” teve um desempenho expressivamente menor que os demais, com 56,8%.

**Tabela 2:** Percentual de desempenho dos serviços de atenção primária à saúde na dimensão “prevenção da sífilis congênita na atenção primária à saúde” e domínios subsequentes, São Paulo, 2017 (n = 2.565).

Agrupamentos de indicadores	Mediana	Média	DP	IC95% (Média)
Dimensão “prevenção da sífilis congênita na atenção primária à saúde”	76,8	74,9	14,3	74,3–75,4
Domínios				
Infraestrutura e recursos básicos	80,0	79,5	15,2	78,9–80,1
Ações educativas e prevenção de IST	57,1	56,8	29,4	55,6–57,9
Diagnóstico e tratamento da sífilis adquirida	100	89,8	17,7	89,1–90,4
Prevenção da sífilis congênita no pré-natal	71,4	73,3	17,5	72,6–73,9

IST: infecções sexualmente transmissíveis; DP: desvio padrão; IC95%: intervalo de confiança de 95%.

Com base na distribuição do desempenho do conjunto dos serviços na dimensão “prevenção da sífilis congênita na APS”, foram construídos grupos de qualidade para analisar a variação no desempenho entre os serviços ( Tabela 3 ). O G4 reuniu aqueles serviços que tiveram maior desempenho (≥ 86,4%), seguidos de G3 (77,1–85,7%) e G2 (67,1–76,8%). Os serviços com menor desempenho foram reunidos no G1 (≤ 66,4%).

**Tabela 3:** Grupos de qualidade ( *clusters* ) na dimensão “prevenção da sífilis congênita na atenção primária à saúde” por frequência e porcentagem de serviços, intervalo de percentual de desempenho e medidas de tendência central e dispersão (n = 2.565).

Grupos*	n	%	Desempenho mínimo	Desempenho máximo	Média	Mediana	DP
G1	675	26,3	8,6	66,4	55,9	58,9	10,0
G2	609	23,7	67,1	76,8	72,5	73,2	2,8
G3	683	26,6	77,1	85,7	81,3	80,7	2,6
G4	598	23,3	86,4	100	91,4	90,4	3,7
Total	2.565	100	-	-	-	-	-

DP: desvio padrão.

*Quartil; DP: desvio padrão.

Considerando os grupos de qualidade, realizou-se uma comparação entre os quatro grupos para cada indicador utilizado na matriz avaliativa ( Tabela 4 ). Segundo o teste qui-quadrado, observou-se uma diferença significativa entre os grupos de qualidade para todos os indicadores. Com o intuito de identificar quais grupos diferiram entre si, foi aplicado o teste Z, mostrando que a proporção de quase todos os indicadores se diferencia entre os quatro grupos, com algumas exceções: nos indicadores com frequências maiores no conjunto de serviços, a proporção por grupo tende a ter semelhanças em G2, G3 e G4 – com variações entre eles – porém, o grupo com menor qualidade (G1) sempre se diferencia dos demais. Em apenas dois indicadores (ausência de casos de sífilis congênita e disponibilidade de preservativo feminino), G1 se assemelha a G2.

**Tabela 4:** Comparação da frequência de cada indicador pelos quatro grupo de qualidade, segundo teste qui-quadrado e testes Z (n = 2.565).

Indicadores	Grupos de qualidade ( *clusters* )								p^**^

	G1		G2		G3		G4		
			
	n^*^	%	n^*^	%	n^*^	%	n^*^	%	
Domínio: Infraestrutura e recursos básicos									
Disponibilidade de preservativo masculino na unidade	661^a^	97,9	606^b^	99,5	681^b^	99,7	597^b^	99,8	< 0,001
Disponibilidade de enfermeiro fixo na unidade	641^a^	95,0	603^b^	99,0	673^b^	98,5	594^b^	99,3	< 0,001
Acesso a serviços de referência em IST, aids e hepatites virais	526^a^	77,9	533^b^	87,5	622^c^	91,1	579^d^	96,8	< 0,001
Disponibilidade de clínico geral e/ou médico de família fixo na unidade	521^a^	77,2	517^b^	84,9	618^c^	90,5	569^d^	95,2	< 0,001
Disponibilidade de preservativo feminino na unidade	544^a^	80,6	515^a,b^	84,6	600^b^	87,8	563^c^	94,1	< 0,001
Oferta de teste rápido para sífilis na rotina da unidade	439^a^	65,0	521^b^	85,6	622^c^	91,1	584^d^	97,7	< 0,001
Coleta de material para exames laboratoriais (sangue e urina) como procedimento de rotina na unidade	423^a^	62,7	461^b^	75,7	541^b^	79,2	519^c^	86,8	< 0,001
Aplicação da penicilina benzatina (Benzetacil) como procedimento de rotina na unidade	345^a^	51,1	437^b^	71,8	551^c^	80,7	548^d^	91,6	< 0,001
Participação em estratégias de formação e/ou educação permanente sobre IST e aids no último ano	246^a^	36,4	374^b^	61,4	506^c^	74,1	545^d^	91,1	< 0,001
Disponibilidade dos medicamentos para o tratamento de IST mais utilizados	126^a^	18,7	198^b^	32,5	302^c^	44,2	343^d^	57,4	< 0,001
Ações educativas e prevenção de IST/aids									
Tema IST/aids abordado em ações de educação em saúde realizadas na unidade no último ano	319^a^	47,3	458^b^	75,2	605^c^	88,6	588^d^	98,3	< 0,001
Orientações sobre sexualidade e prevenção de IST/aids incluídas em ações para atenção ao adolescente desenvolvidas na comunidade	177^a^	26,2	288^b^	47,3	487^c^	71,3	563^d^	94,1	< 0,001
Ações que abordam IST, aids e hepatites virais desenvolvidas com regularidade na atenção ao adulto	334^a^	49,5	487^b^	80,0	643^c^	94,1	588^d^	98,3	< 0,001
Orientações sobre sexualidade e prevenção de IST/aids incluídas na rotina da atenção ao adolescente na unidade	198^a^	29,3	334^b^	54,8	559^c^	81,8	580^d^	97,0	< 0,001
Pessoas que fazem uso abusivo de álcool e outras drogas como público-alvo de ações programáticas de prevenção de IST/aids realizadas pela unidade	52^a^	7,7	149^b^	24,5	359^c^	52,6	526^d^	88,0	< 0,001
Prevenção de IST/aids abordada em ações de educação em saúde realizadas na comunidade no último ano	118^a^	17,5	162^b^	26,6	305^c^	44,7	429^d^	71,7	< 0,001
Grupos mais vulneráveis (caminhoneiros, profissionais do sexo, entre outros) como público-alvo das ações programáticas de prevenção de IST/aids realizadas pela unidade	34^a^	5,0	117^b^	19,2	274^c^	40,1	461^d^	77,1	< 0,001
Diagnóstico e tratamento da sífilis adquirida									
Convocação de usuário em caso de resultado positivo em sorologia para sífilis	576^a^	85,3	589^b^	96,7	670^b,c^	98,1	594^c^	99,3	< 0,001
Orientação sobre diagnóstico e tratamento em caso de resultado positivo em sorologia para sífilis	546^a^	80,9	587^b^	96,4	673^c^	98,5	598^d^	100	< 0,001
Preenchimento da ficha de notificação compulsória em casos de sífilis	538^a^	79,7	585^b^	96,1	658^b^	96,3	587^c^	98,2	< 0,001
Realização de teste rápido ou sorologia para sífilis em caso sugestivo de IST/aids	431^a^	63,9	558^b^	91,6	656^c^	96,0	592^d^	99,0	< 0,001
Convocação de usuários faltosos com sífilis	364^a^	53,9	528^b^	86,7	630^c^	92,2	590^d^	98,7	< 0,001
Orientação de cuidado com as parcerias em caso de resultado positivo em sorologia para sífilis	557^a^	82,5	588^b^	96,6	672^c^	98,4	595^c^	99,5	< 0,001
Tratamento e seguimento realizado na própria unidade em caso de resultado positivo em sorologia para sífilis	416^a^	61,6	520^b^	85,4	643^c^	94,1	587^d^	98,2	< 0,001
Prevenção da sífilis congênita no pré-natal									
Realização de seis ou mais consultas de pré-natal	611^a^	90,5	582^b^	95,6	667^c^	97,7	591^c^	98,8	< 0,001
Convocação de gestantes faltosas	531^a^	78,7	581^b^	95,4	665^b^	97,4	593^c^	99,2	< 0,001
Proporção média de gestantes com pré-natal iniciado no primeiro trimestre superior a 80%	446^a^	66,1	462^b^	75,9	544^b^	79,6	534^c^	89,3	< 0,001
Solicitação de teste rápido ou sorologia para sífilis no primeiro e terceiro trimestre gestacional	339^a^	50,2	444^b^	72,9	503^b^	73,6	505^c^	84,4	< 0,001
Oferta de tratamento da sífilis para gestante e parceria na unidade	363^a^	53,8	464^b^	76,2	588^c^	86,1	579^d^	96,8	< 0,001
Realização do pré-natal do homem	193^a^	28,6	265^b^	43,5	386^c^	56,5	450^d^	75,3	< 0,001
Ausência de casos de sífilis congênita nos últimos três anos	285^a^	42,2	279^a^	45,8	352^b^	51,5	361^c^	60,4	< 0,001

IST: infecções sexualmente transmissíveis.

*Testes Z.

**Teste qui-quadrado.

Letras subscritas diferentes indicam proporções estatisticamente distintas.

O cruzamento entre os quatro grupos de qualidade e os tipos de arranjos organizacionais (USF, UBS tradicional, UBS com PACS/ESF e outros) mostrou uma diferença significativa no teste qui-quadrado (p < 0,001). Em relação às USF, o grupo de melhor qualidade (G4) foi o que apresentou maior proporção desses serviços na sua composição (G4 = 56,7%; G3 = 49,6%; G2 = 45,6%; G1 = 37,6%), sendo que quase todos os grupos se diferenciaram entre si, exceto G2 e G3, que se assemelharam, segundo teste Z.

No caso das UBS tradicionais, o grupo de menor qualidade (G1) foi o que apresentou maior proporção (G1 = 35%; G2 = 25,6%; G3 = 18,7%; G4 = 9,7%), com todos os grupos se diferenciando entre si. As unidades mistas, embora se distribuam heterogeneamente entre os quatro grupos, também apresentam maior proporção no grupo de maior qualidade (G4 = 31,4%; G3 = 28,4%; G2 = 25,6%; G1 = 23,6%). Vale destacar que, ao comparar os grupos de qualidade e as variáveis de localização dos serviços, o teste qui-quadrado não mostrou associação significativa.

## DISCUSSÃO

A avaliação realizada, ainda que baseada em dados descritivos, permitiu identificar alguns dos principais pontos do processo de trabalho que necessitam de qualificação para o fortalecimento da prevenção da sífilis congênita na APS paulista, como o rastreamento da sífilis no pré-natal, o tratamento adequado da sífilis gestacional e adquirida com o uso de penicilina benzatina e a incorporação de ações de educação em saúde na unidade e no território.

O desempenho médio dos serviços avaliados revela que, apesar de a prevenção da sífilis congênita contar com ações que pertencem à rotina de trabalho da APS, ainda são muitos os serviços que falham em cumprir adequadamente as recomendações estabelecidas por protocolos e diretrizes, conforme apontado por outros estudos^15 , 16^. Embora os dados se refiram a um inquérito realizado em 2017, as taxas de incidência de sífilis congênita no estado de São Paulo^5^indicam a provável persistência dos problemas identificados. As mudanças que foram introduzidas a partir da pandemia da covid-19 alteraram a rotina dos serviços de APS, comprometendo ações programáticas, como o pré-natal e a vigilância em saúde, o que pode ter contribuído para a manutenção dos pontos identificados em 2017^17 , 18^.

Os protocolos brasileiros que abordam a prevenção da sífilis congênita enfatizam a importância do pré-natal, uma vez que é durante o seguimento da gestante na APS que são desenvolvidas ações que possibilitam o diagnóstico precoce da infecção materna e seu tratamento oportuno^4 , 6^. Todavia, observa-se na literatura que, entre os casos notificados de sífilis congênita, uma grande parcela das mães realizou o pré-natal^16 , 19 , 20^, indicando que o simples acesso às consultas não tem garantido a redução da taxa de incidência^21^.

O desafio da prevenção da transmissão vertical da sífilis no Brasil está centrado, principalmente, no comprometimento da qualidade do cuidado ofertado. Os resultados aqui apresentados trazem dados que corroboram essa hipótese, uma vez que o domínio “prevenção da sífilis congênita na atenção pré-natal” teve o segundo menor desempenho, ou seja, mesmo em ações priorizadas exaustivamente pelos protocolos, como o rastreamento e tratamento precoce da sífilis gestacional, existem lacunas importantes tanto em relação à oferta como na disponibilização de insumos para sua realização.

O rastreamento da sífilis é preconizado em dois momentos durante o acompanhamento pré-natal na APS: no primeiro e no terceiro trimestre gestacional^2^. Entre os serviços analisados, poucos seguiam essa recomendação (69,8%), limitando-se à solicitação de apenas uma testagem para sífilis durante o primeiro trimestre (95,3%). Isso destaca a dificuldade dos serviços em adequar sua rotina às recomendações e até mesmo a falta de conhecimento dos profissionais e gestores quanto a protocolos, impacto e custo-benefício do rastreamento adequado^22^.

Protocolos e diretrizes clínicas atuam como ferramentas essenciais para direcionar e respaldar as práticas realizadas pelos serviços de saúde, mas somente sua existência não garante a qualidade do cuidado. Fatores que envolvem a organização das unidades, como o modelo de gestão e, principalmente, o processo de trabalho operacionalizado, podem trazer limitações técnico-operacionais, prejudicando a qualidade e a efetividade da assistência à saúde^23^.

O teste rápido para sífilis é uma das estratégias de rastreamento que recebe destaque como recurso de fácil execução e alto custo-efetividade. Quando realizado logo no primeiro atendimento à gestante, propicia diagnóstico precoce e tratamento oportuno da infecção materna^2^. Essa tem sido uma ferramenta utilizada por grande parte dos serviços estudados e necessita de contínuo fortalecimento e incentivo à sua execução.

As dificuldades na prevenção da sífilis congênita na APS se estendem à problemática do uso da penicilina benzatina como medicamento de primeira escolha no tratamento da sífilis gestacional, muitas vezes sustentada pela falta de conhecimento dos profissionais sobre os esquemas terapêuticos mais adequados^22^e pelo medo das reações adversas de sua aplicação^24^.

Trata-se do único medicamento capaz de atravessar a barreira placentária e chegar até o feto, portanto, as fragilidades existentes na sua oferta como um procedimento de rotina para tratamento de gestantes e parcerias, colocam em risco o acesso a um tratamento seguro, eficaz e oportuno da sífilis na gestação^3^. Essa dificuldade é sustentada neste estudo, com indicadores mostrando que uma parcela significativa das unidades não oferece o tratamento a gestantes e parcerias e não aplica penicilina benzatina como procedimento de rotina.

Apesar do desempenho insuficiente expresso pelos indicadores de tratamento, é importante ressaltar que houve avanços. Ao comparar resultados deste estudo com o desenvolvido por Sanine et al.^15^, observa-se melhor desempenho nos indicadores relacionados à aplicação da penicilina benzatina na unidade e na oferta de tratamento para gestantes e parcerias, em 2017 comparado a 2010. Essa melhoria possivelmente reflete os esforços de capacitação e medidas técnicas empreendidas tanto pelo Ministério da Saúde^6 , 25 , 26^como pela Secretaria de Estado da Saúde de São Paulo^27^.

Para além da atenção ao pré-natal, o modelo de avaliação da prevenção da sífilis congênita apresentado considera a importância de práticas que favorecem a interrupção da cadeia de transmissão da sífilis em outras fases da vida por meio de ações de prevenção, diagnóstico precoce e manejo da sífilis em mulheres em idade fértil e em suas parcerias sexuais.

Os serviços avaliados possuem um bom desempenho relacionado à atenção às IST, sendo que o domínio “diagnóstico e tratamento da sífilis adquirida” foi o que obteve maior média entre os serviços, e nenhum de seus indicadores teve uma frequência menor que 80%. Tal resultado pode ser reflexo da tradição da assistência às IST na APS paulista, uma vez que o estado foi pioneiro na organização de respostas da rede à epidemia de aids no país e, desde então, assume forte papel na linha de prevenção e assistência a IST/aids, empreendendo iniciativas que têm o objetivo de fortalecer a integração dessas ações^27 , 28^.

Na busca pela eliminação da sífilis congênita, é importante que ações educativas sejam realizadas junto à comunidade, às gestantes e às famílias, com o objetivo de aumentar o conhecimento desses atores sobre a saúde materno-infantil e garantir uma maior autonomia no cuidado^4^. Entretanto, o domínio “ações educativas e prevenção de IST” foi o que obteve menor desempenho, com frequências baixas em todos os indicadores, especialmente nos que abordam práticas de educação em saúde desenvolvidas na comunidade e que envolvem grupos em situação de vulnerabilidade. Esses resultados corroboram a literatura, que indica que as práticas de prevenção às IST/aids e de educação em saúde ocupam um lugar secundário no processo de trabalho em saúde^10 , 29^.

As médias e medianas altas e semelhantes entre os grupos de qualidade aponta a ocorrência de um pequeno intervalo percentual entre eles, com exceção de G1, indicando que cerca de 70% dos serviços tiveram desempenho médio acima de 70%. Entretanto, para uma doença evitável por meio de diversas práticas desenvolvidas nos serviços de APS em diferentes momentos, esse desempenho pode ser considerado insuficiente.

Todos os indicadores incluídos na matriz avaliativa foram capazes de diferenciar os grupos entre si, fato que estatisticamente pode ser justificado pelo grande número de serviços da amostra (2.565), mas, por outro lado, reflete a diferenciação entre os serviços e a capacidade da matriz proposta em identificar atributos distintos que qualificam a prevenção da sífilis congênita.

O melhor desempenho de unidades organizadas segundo as diretrizes de saúde da família está bem demonstrado na literatura^30^, e a avaliação realizada reafirma essa tendência. Por outro lado, é fato que mesmo as USF, e as UBS que possuem ESF ou PACS integradas, também requerem um maior investimento para que efetivem uma atenção integral, visto que muitas dessas unidades também apresentaram baixo desempenho.

Um dos limites deste trabalho é o poder de generalização dos resultados, uma vez que se referem apenas aos serviços participantes. Outro limite diz respeito ao uso de dados secundários oriundos de um inquérito que avaliou o conjunto de ações da APS, sendo a prevenção da sífilis congênita um recorte desse conjunto. Além disso, estudos transversais descritivos têm limites dados pelo próprio recorte temporal que fazem da realidade. Entretanto, a avaliação de desempenho de serviços aponta questões que exigem mudanças processuais e medidas de planejamento e gestão, contribuindo para a melhoria da qualidade.

As fragilidades identificadas na organização dos serviços têm grande governabilidade local e apontam prioridades que precisam ser incorporadas e que representam a consolidação de políticas e diretrizes já disponíveis. Destaca-se a necessidade de investimentos na capacidade técnica dos serviços e na qualificação profissional para execução dos protocolos de maneira efetiva.

Os resultados confirmam a importância da qualificação do pré-natal, já apontada em outros estudos, e evidenciam o valor da realização de avaliações que induzem reflexões nas equipes sobre a organização dos processos de trabalho e que, assim, contribuem para a instauração de processos de mudança que produzam impacto na prevenção da sífilis congênita.
